# Age-related changes in microRNA levels in serum

**DOI:** 10.18632/aging.100603

**Published:** 2013-09-27

**Authors:** Nicole Noren Hooten, Megan Fitzpatrick, William H. Wood, Supriyo De, Ngozi Ejiogu, Yongqing Zhang, Julie A. Mattison, Kevin G. Becker, Alan B. Zonderman, Michele K. Evans

**Affiliations:** ^1^ Laboratory of Epidemiology and Population Sciences, National Institutes of Health, Baltimore, MD 21224, USA; ^2^ Laboratory of Genetics, National Institutes of Health, Baltimore, MD 21224, USA; ^3^ Translational Gerontology Branch, National Institute on Aging, National Institutes of Health, Baltimore, MD 21224, USA

**Keywords:** circulating, miRNA, noncoding RNA, age, aging, biomarker, exRNA, extracellular RNA

## Abstract

microRNAs (miRNAs) are small noncoding RNAs that post-transcriptionally regulate gene expression by targeting specific mRNAs. Altered expression of circulating miRNAs have been associated with age-related diseases including cancer and cardiovascular disease. Although we and others have found an age-dependent decrease in miRNA expression in peripheral blood mononuclear cells (PBMCs), little is known about the role of circulating miRNAs in human aging. Here, we examined miRNA expression in human serum from young (mean age 30 years) and old (mean age 64 years) individuals using next generation sequencing technology and real-time quantitative PCR. Of the miRNAs that we found to be present in serum, three were significantly decreased in 20 older individuals compared to 20 younger individuals: miR-151a-5p, miR-181a-5p and miR-1248. Consistent with our data in humans, these miRNAs are also present at lower levels in the serum of elderly rhesus monkeys. In humans, miR-1248 was found to regulate the expression of mRNAs involved in inflammatory pathways and miR-181a was found to correlate negatively with the pro-inflammatory cytokines IL-6 and TNFα and to correlate positively with the anti-inflammatory cytokines TGFβ and IL-10. These results suggest that circulating miRNAs may be a biological marker of aging and could also be important for regulating longevity. Identification of stable miRNA biomarkers in serum could have great potential as a noninvasive diagnostic tool as well as enhance our understanding of physiological changes that occur with age.

## INTRODUCTION

Aging is a highly complex process where over time the accumulation of cellular and molecular damage leads to the functional decline of tissues and organs that eventually increase disease susceptibility and mortality. Although aging can be influenced by environmental factors, recent data have emerged that genetic factors also play a definitive role in regulating lifespan. In particular, modulation of gene expression in model organisms has been shown to affect longevity [1-3]. miRNAs are short (18-22 nucleotides), noncoding RNAs that bind to target sequences in mRNA, typically resulting in post-transcriptional alteration of mRNA stability or translation efficiency. microRNAs are key regulators of gene expression that affect a wide range of cellular processes including differentiation, proliferation, apoptosis, and replicative senescence [4-6]. It is estimated that approximately 60% of human genes are regulated by miRNAs [5, 7, 8], suggesting that these small RNAs play important roles in a variety of biological processes.

miRNAs have been well-studied in the *Caenorhabditis elegans* model system, where miRNA expression changes with organismal lifespan and individual miRNAs have been shown to modulate lifespan and to predict individual longevity [9-12]. With increasing organismal complexity, the precise role of miRNAs in aging and lifespan is less understood [2, 3, 7]. It has been shown that miRNAs are differentially expressed with age in mouse brain, liver and skeletal muscle and in the long-lived Ames dwarf mouse; however, the expression patterns appear to be tissue specific [2, 7, 13]. In humans, we showed previously that miRNA expression changes with human age in peripheral blood mononuclear cells (PBMCs) [14]. Specifically we found that the majority of miRNAs are downregulated with age and 9 age-associated miRNAs significantly decreased in abundance in older individuals (mean age 64) compared to young individuals (mean age 30). Since then, several reports have shown miRNA expression changes in centenarians compared to either adults or octogenarians [15-17].

miRNAs also circulate in a cell-free form in body fluids including serum and plasma [18-20]. These cell-free miRNAs are highly stable and resistant to harsh conditions including heat, pH changes, freeze/thaw cycles and extended storage [18-20]. Currently, two major theories persist as to the origin of circulating miRNAs. The first posits that miRNAs are passively released into circulation during tissue injury. The second proposes that cell-free miRNAs protected from RNase activity by microvesicles, exosomes, or RNA-binding proteins are shed from the plasma membranes of various cell types [18-20].

Altered expression of miRNAs in serum has been associated with several types of cancer including squamous cell, gastric, lung, colorectal and prostate [18, 20]. Circulating miRNAs are also thought to play a role in the development and progression of cardiovascular disease [21]. For example, miR-150 was found to be reduced in serum of patients with arterial fibrillation and miR-1, miR-134, miR-186, miR-208, miR-233 and miR-499 were all found to be significantly upregulated in serum from acute myocardial infarction (AMI) patients [22-24]. These studies underscore the potential of using serum miRNAs as diagnostics and possibly prognostic markers for various cancers and cardiovascular diseases.

Here, we have analyzed miRNA expression in serum from young and old individuals. Three serum miRNAs were significantly decreased in old individuals: miR-151a-3p, miR-181a-5p and miR-1248. Interestingly, we also found that the majority of serum miRNAs decrease in abundance with age in rhesus monkeys. In addition, we employed a combinatory approach using bioinformatics, serum cytokine screens and microarray to get a broad view of the genes and pathways that may be targeted by these age-associated serum miRNAs. These findings provide insights into the molecular mechanisms underlying the aging process and suggest that serum miRNAs may be used as biomarkers of human age.

## RESULTS

### miRNA expression in young and old individuals using deep gene sequencing

**Figure 1:**
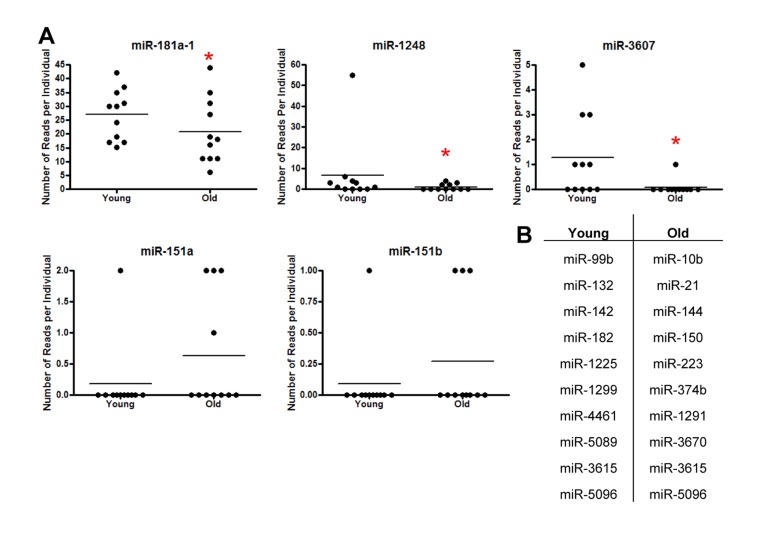
Identification of miRNAs in serum from young and old individuals **(A)** Frequency of miRNA reads per individual using small RNA NGS from 11 young (~30 yr) and 11 old (~64 yr) individuals for miR-181a-1, miR-3607, miR-1248, miR-151a and miR-151b. Horizontal lines represent average number of reads. miR-181a, miR-1248 and miR-3607 were significantly downregulated in serum from old individuals using Poisson regression (miR-181a-1 P=0.03, miR-3607 P=0.01, miR-1248 P=4.9×10^−9^). **(B)** miRNAs identified that had only one read per individual.

In order to study age-related changes in miRNA expression in human serum, we used Illumina small-RNA next generation sequencing (NGS) technology. We obtained serum from 11 young and 11 old individuals selected from a sub-cohort of the Healthy Aging in Neighborhoods of Diversity across the Lifespan (HANDLS) study. These participants were used in our previous examination of miRNA expression changes with age in PBMCs [14]. Demographic information for these participants is presented in Table 1. Interestingly, miRDeep2 software, which aligns sequences to both mature and precursor miRNAs, revealed that the majority (87%) of miRNA sequences in human serum were precursors. miRDeep2 detected 23 miRNAs in our samples, five miRNAs of which were found to be present in the serum from both young and old individuals (Figure 1A). Importantly, miR-181a-1, miR-1248 and miR-3607 were significantly lower in old individuals (Figure 1A). Several miRNAs had only one read in one individual and thus were excluded from further analysis (Figure 1B).

**Table 1:** Participant demographic information

**A) Sequencing**	Young	Old
N	11	11
Age	30.5±0.5	64.6±0.5
Sex	6 F, 5 M	5 F, 6 M
Race	8 W, 3 AA	6 W, 5 AA
Sex and Race	5 W-F, 1 AA-F 3 W-M, 2 AA-M	3 W-F, 2 AA-F 3 W-M, 3 AA-M
**B) Validation**	Young	Old
N	20	20
Age	30.6±0.5	64.4±0.5
Sex	12 F, 8 M	12 F, 8 M
Race	10 W, 10 AA	12 W, 8 AA
Sex and Race	7 W-F, 5 AA-F 3 W-M, 5 AA-M	6 W-F, 6 AA-F 6 W-M, 2 AA-M

Demographics for individuals in this sub-cohort of the HANDLS study. Age is reported as the mean ± SD. Abbreviations are as follows: W, White; AA, African American; F, Female; M, Male.

### Validation of miRNA expression changes in young versus old individuals

To validate our sequencing results, we employed real-time RT-PCR using sequence-specific miRNA primers to analyze the expression levels of the five miRNAs with the highest number of sequencing reads in our original cohort (n=11 for each group). In addition, we expanded this initial cohort to include more young (n=20; mean age 30.6) and old (n=20; mean age 64.4) individuals. Demographic information for this validation cohort is in Table 1. Three of the miRNAs we analyzed, miR-151a, miR-181a-1 and miR-3607, are precursors that can be processed into two mature miRNAs according to miRBase (miR-151a-3p and −5p, miR-181a-3p and −5p and miR-3607-3p and −5p). Therefore, in our validation experiments, we tested both 3p and 5p mature sequences and also have referred to these miRNAs using the nomenclature in miRBase. In addition, miR-151b has the same sequence as miR-151a-5p and thus, we considered these miRNAs to be the same.

**Figure 2:**
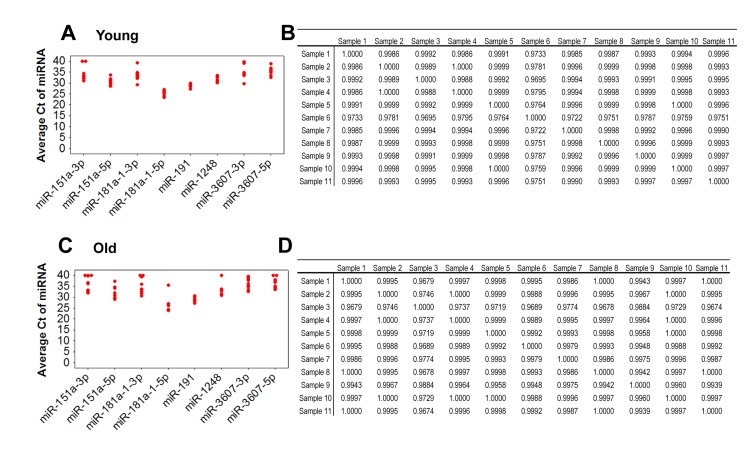
Comparison of expression variance of 8 miRNAs in 11 young and 11 old individuals by real-time RT-PCR RNA isolated from serum was reverse transcribed and real-time RT-PCR was performed with miRNA specific primers. Data was normalized to miR-191. C_t_ values of each miRNA for each young (**A**) and old (**C**) participant. C_t_ values were used for analysis of the Pearson correlation coefficient of miRNA expression between young participant samples (**B**) and between old participant samples (**D**). R values are indicated and a value of 1 indicates perfect correlation.

In order to evaluate the variation of miRNA expression among our initial cohort of 11 young and 11 old participants, we analyzed the expression of eight miRNAs in serum using reverse transcription (RT) followed by real-time, quantitative (q)PCR. Expression levels among young and old individuals were consistent with a Pearson's correlation coefficient (r) close to 1 for the all samples (Figure 2). Similar data were also observed with the entire cohort of 40 (data not shown). Comparison of the C_t_ values for these miRNAs showed low variability among the young and old groups (Figure 2). These data suggest that miRNA expression is reproducibly consistent among young individuals and among old individuals.

**Figure 3:**
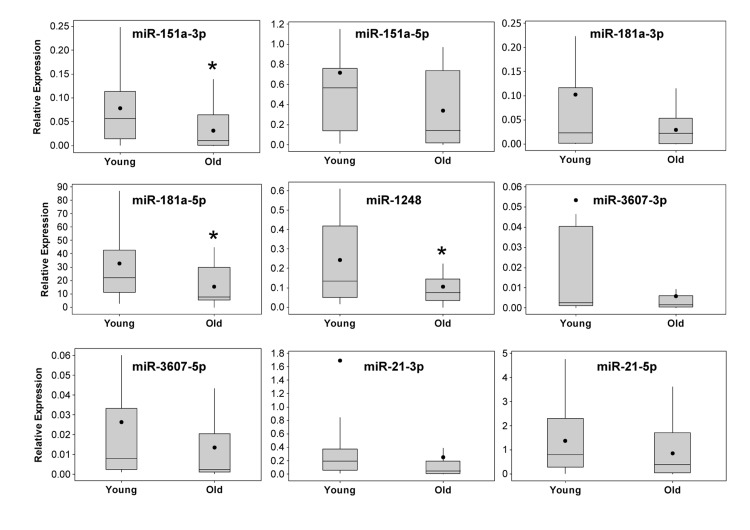
Real-time RT-PCR validation of miRNA expression in serum from young and old participants The expression levels of miRNAs identified by sequencing analysis were validated in 20 young and 20 old participant serum samples (see Table 1 for demographic information) using real-time RT-PCR. Sequence specific primers for the indicated miRNAs were used and the data was normalized to miR-191. Box and whisker plots for each miRNA are shown. Whiskers represent ± the standard deviation, the box extends to upper and lower quartiles, lines represent the median and closed circles represent the mean. *P<0.05 comparing young and old by Student's t-test.

Through this analysis, we observed differences in miRNA abundance between young and old individuals for several miRNAs. miR-181a-5p and miR-1248 were significantly decreased in old individuals (Figure 3). In addition, although our sequencing results showed increased levels of miR-151a/b in old individuals, our RT-qPCR analysis in the larger cohort of individuals showed that miR-151a-3p is significantly lower in old individuals. Lower levels of miR-151a-5p, miR-181a-1-3p, miR-3607-3p and miR-3607-5p were also observed in old individuals, though not significantly. Consistent with our sequencing data, we found that miR-181a-5p was by far the most highly expressed serum miRNA among the miRNAs that we analyzed (Figure 3), confirming that our sequencing data gave a quantitative assessment of miRNA abundance. In addition, the expression of many of these age-associated miRNAs was highly correlated (Figure 2 and Table 2). Given the recent data that serum miR-21 increases in abundance with age [25], we also examined miR-21 levels in young and old individuals. miR-21-3p expression was not different between our age groups and miR-21-5p levels decreased, albeit not significantly, in our older cohort. These differences are likely due to the significant differences in the age span of the young and old cohorts for the Olivieri et al. study. The young cohort in that study ranged in age from 24-64 which encompasses both our young and old cohort while their old cohort is enriched with significantly aged individuals, including 30 centenarians.

**Table 2:** Correlations of the different miRNAs, cytokines and age

	**age**	**21-3p**	**21-5p**	**151a-3p**	**151a-5p**	**181a-3p**	**181a-5p**	**1248**	**3607-5p**	**3607-3p**	**IL-6**	**TGFβ2**	**TNFα**
**age**	1.00	−0.17	−0.21	−0.34*	−0.29	−0.21	−0.33*	−0.31*	−0.20	−0.19	0.65***	−0.12	0.26
**21-3p**	−0.17	1.00	0.33*	0.11	0.07	0.95***	0.41**	0.28	0.36*	0.99***	−0.32*	0.90***	−0.16
**21-5p**	−0.21	0.33*	1.00	0.84***	0.53***	0.40**	0.42**	0.59***	0.48**	0.37*	−0.24	0.34*	−0.37*
**151a-3p**	−0.34*	0.11	0.84***	1.00	0.55***	0.23	0.37*	0.61***	0.39*	0.17	−0.36*	0.15	−0.38*
**151a-5p**	−0.29	0.07	0.53***	0.55***	1.00	0.14	0.87***	0.68***	0.73***	0.08	−0.32*	0.08	−0.34*
**181a-3p**	−0.21	0.95***	0.40**	0.23	0.14	1.00	0.44**	0.36*	0.39*	0.97***	−0.35*	0.87***	−0.22
**181a-5p**	−0.33*	0.41**	0.42**	0.37*	0.87***	0.44**	1.00	0.74***	0.80***	0.41**	−0.35*	0.36*	−0.34*
**1248**	−0.31*	0.28	0.59***	0.61***	0.68***	0.36*	0.74***	1.00	0.74***	0.32*	−0.28	0.24	−0.31
**3607-5p**	−0.20	0.36*	0.48**	0.39*	0.73***	0.39*	0.80***	0.74***	1.00	0.39*	−0.26	0.36*	−0.18
**3607-3p**	−0.19	0.99***	0.37*	0.17	0.08	0.97***	0.41**	0.32*	0.39*	1.00	−0.33*	0.90***	−0.18
**IL-6**	0.65***	−0.32*	−0.24	−0.36*	−0.32*	−0.35*	−0.35*	−0.28	−0.26	−0.33*	1.00	−0.23	0.27
**TGFβ2**	−0.12	0.90***	0.34*	0.15	0.08	0.87***	0.36*	0.24	0.36*	0.90***	−0.23	1.00	−0.19
**TNFα**	0.26	−0.16	−0.37*	−0.38*	−0.34*	−0.22	−0.34*	−0.31	−0.18	−0.18	0.27	−0.19	1.00

R value is indicated and *P<0.05, **P<0.01, ***P<0.001. Power values were used for analysis with the various miRNAs. No significant correlations were observed with race or sex; therefore, these variables are not shown.

### Age-dependent changes in miRNA expression in non-human primates

**Figure 4:**
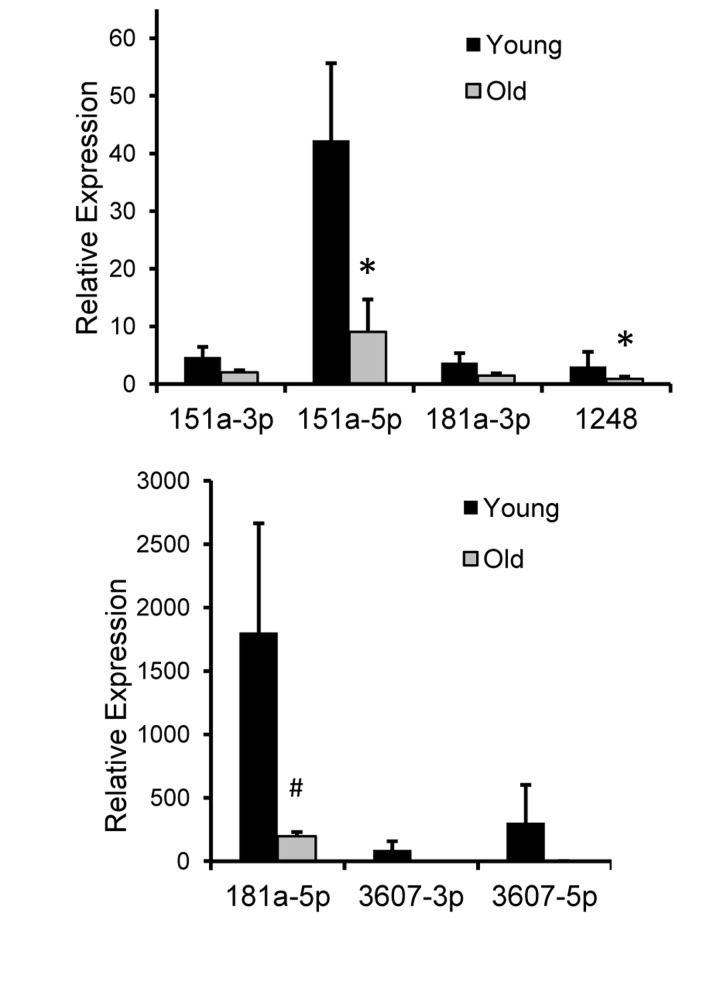
Relative expression of 7 miRNAs in rhesus monkey serum RNA was isolated from serum from ten young male monkeys (mean age = 7.7 yrs) and ten old male monkeys (mean age = 21.7 yrs). Real-time RT-PCR was performed with miRNA specific primers and normalized to miR-191. The histogram represents the ±SEM. *P<0.05 and #P=0.09 using Student's t-test.

We wanted to assess whether the miRNAs that we found in human serum were also expressed in other long-lived species that are used as models of human aging [26]. Therefore, we examined levels of these seven miRNAs in the serum of 10 young (mean age = 7.7±1.5 years) and 10 old (mean age = 21.7±2.1) rhesus monkeys (*Macaca mulatta*). As rhesus monkeys age at approximately three times the rate of humans, the young and old groups of monkeys correspond approximately with the average ages of our young and old human cohort, ~23 for young and ~65 for old. Importantly, we found that all the miRNAs we found expressed in human serum were also present in monkey serum (Figure 4). In addition, the expression levels of these miRNAs are fairly consistent between monkeys and humans in that miR-181a-5p is highly expressed, while other miRNAs are expressed at lower levels. We found significantly lower levels of miR-151-5p and miR-1248 and a close to significant (p=0.09) decrease in miR-181a-5p levels with monkey age (Figure 4).

### Target and Pathway Analysis for serum age-associated miRNAs

**Figure 5:**
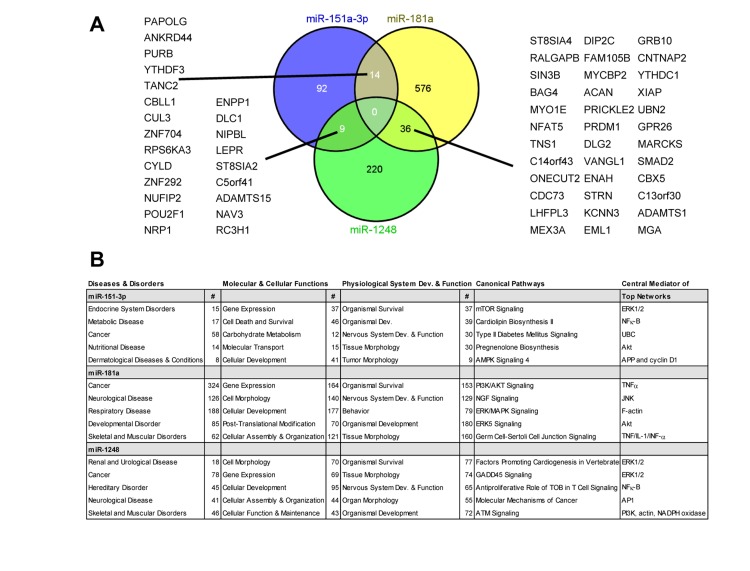
Predicted targets and pathways for the indicated age-associated miRNAs Predicted targets for the 3 miRNAs were obtained using TargetScan software and the overlapping targets were visualized using a Venn diagram (**A**). The overlapping targets are indicated. (**B**) The predicted targets from TargetScan for each miRNA were used for Ingenuity Pathway Analysis. The top five pathways for each parameter are listed and where applicable the number of genes (#) for the particular pathway. For each parameter listed, P<0.05. Dev.=development.

To understand more thoroughly how these miRNAs may contribute to the aging process, we examined several mRNA targets of miR-151a-3p, miR-181a-5p and miR-1248 as predicted using TargetScan 6.2. We identified 115, 626 and 265 mRNAs that were predicted to be targeted by miR-151a-3p, miR-181a and miR-1248, respectively (Figure 5A). No predicted targets overlapped between the 3 different miRNAs, however, there was some overlap between the different combinations of 2 miRNAs (Figure 5A). In addition, we used the targets from TargetScan to input into Ingenuity Pathway Analysis to reveal the top 5 different diseases and disorders, molecular and cellular functions, physiological system development and function, and canonical pathways associated with each miRNA (Figure 5B). We also performed network analysis and listed the central mediator for each of the top five networks (Figure 5B).

Cancer and neurological disease overlapped among all three miRNAs, consistent with the fact that age is a significant risk factor for these two diseases. All three of these miRNAs are predicted to function in regulating gene expression, which is intriguing given that the age-associated miRNAs in our previous study of PBMCs were also predominantly predicted to target gene expression pathways [14]. Several other molecular and cellular functions overlapped between these 3 miRNAs including cellular development, cell morphology, and cellular assembly and organization. Interestingly, organismal survival was the top physiological pathway for each of the miRNAs and several predicted target genes involved in survival pathways overlapped between at least two of the three miRNAs. There was no overlap between the canonical pathways; however, in the predicted top networks, central mediators including ERK1/2, Akt, NF-κB and actin overlapped among these miRNAs. This analysis suggests that miR-151a-3p, miR-181a-5p and miR-1248 may target similar, yet distinct signaling pathways important for the aging process.

Since the top network mediator for miR-181a is TNFα and because this miRNA has been shown to target several other inflammatory markers [27-29], we hypothesized that the decline in miR-181a with age that we found may be a cause of the increased inflammation that occurs with old age. Therefore, we examined several inflammatory markers that have been reported to be targeted by miR-181a in serum from the young and old individuals of our cohort. We found that miR-181a expression was significantly negatively correlated with the pro-inflammatory cytokines IL-6 and TNFα and positively correlated with the anti-inflammatory cytokine TGFβ (Table 2). Various other age-associated serum miRNAs were also correlated with IL-6, TNFα and TGFβ levels (Table 2). Other inflammatory miR-181a targets, IL-1α, IL-1β, IL-8 and LIF, were found to be low or undetectable in the serum from our young and old individuals. Although the anti-inflammatory cytokine IL-10 was only detected in 6 participants (3 young and 3 old), it was highly correlated with miR-181a-5p levels (R=0.84, P<0.01). Low levels of circulating IL-10 is consistent with other reports that this anti-inflammatory marker is induced and not necessarily expressed under non-stimulated conditions [30, 31]. These data suggest that the downregulation of miR-181a we observed with age in serum correlates with an upregulation of pro-inflammatory cytokines and downregulation of anti-inflammatory cytokines.

**Figure 6:**
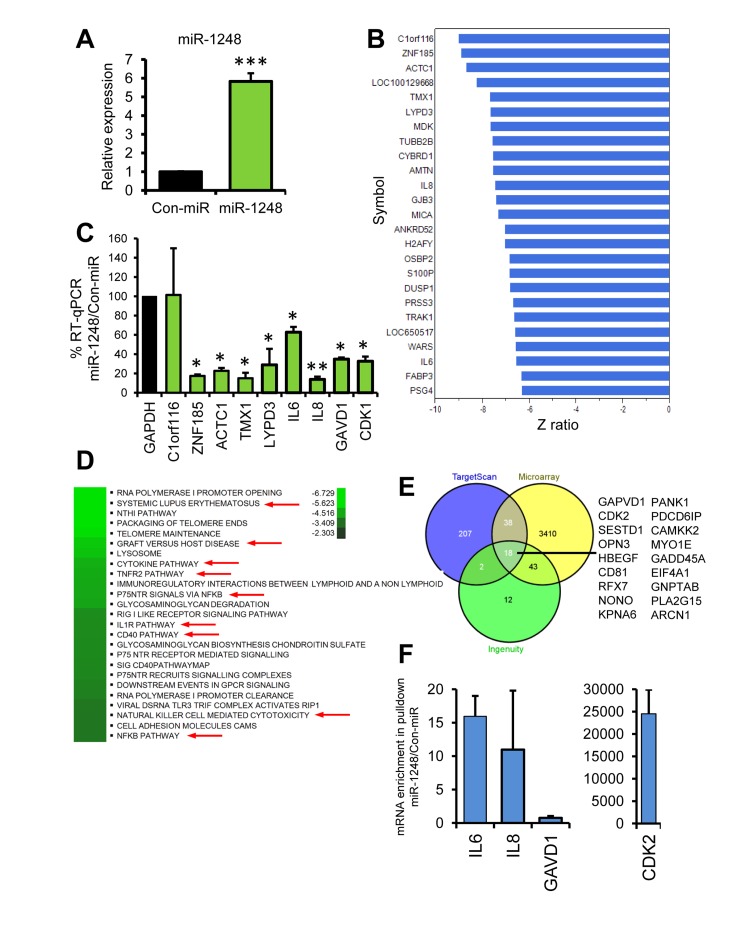
Pathway and Target analysis for miR-1248 (**A**) HeLa cells transfected with Con-miR or miR-1248 were analyzed 48 hrs after transfection for miR-1248 expression using real-time RT-PCR. Data was normalized to U6 expression and the histogram represents the mean + SEM from 3 different experiments. (**B**) Total RNA from (A) was analyzed using genome-wide Illumina microarrays. The top downregulated genes are listed and the corresponding numerical complete dataset is presented in Suppl. Table S1. (**C**) RT-qPCR validation of microarray results using mRNA-specific primer pairs. (**D**) Top canonical pathways for genes downregulated by miR-1248 overexpression are shown on the heatmap. Red arrows indicate inflammatory and cytokine pathways. (**E**) Venn diagram of putative targets from TargetScan, Ingenuity and miR-1248 microarray analysis. Overlapping mRNAs are listed. (**F** Biotinylated miR-1248 or Con-miR were transfected into HeLa cells and were precipitated using streptavidin beads. Associated mRNAs were isolated and gene-specific primers were used for RT-qPCR.

Given that there is very little known about miR-1248, we sought to determine the potential targets and pathways regulated by this miRNA. Therefore, we overexpressed a miR-1248 mimic in HeLa cells and performed microarray analysis in order to determine which genes and pathways are differentially changed by miR-1248 overexpression (Figure 6). This analysis showed that 1740 mRNAs were downregulated and 1770 mRNAs were upregulated by miR-1248 overexpression (Suppl. Table S1). Since most mRNA targets are degraded by miRNAs, we focused on the top downregulated mRNAs from our microarray (Figure 6B). Interestingly, two of these mRNAs are the age-associated cytokines IL6 and IL8. We further validated by real-time RT-PCR that indeed these cytokines are decreased in abundance in miR-1248 overexpressing cells (Figure 6C). In addition, we further validated other genes that were downregulated in our microarray analysis (Figure 6C). Differentially expressed genes and Z ratios were categorized into known canonical pathways by the Parametric Analysis of Gene-set Enrichment (PAGE). The top canonical pathways that were downregulated in miR-1248 overexpressing cells are shown (Figure 6D). All canonical pathways regulated by miR-1248 are shown in the heat map in Supplemental Figure S1. Several cytokine and inflammatory-associated pathways, including NF-κB, are downregulated in miR-1248 overexpressing cells (Figure 6D). Interestingly, DNA repair pathways are upregulated in miR-1248 overexpressing cells (Suppl. Figure S1), suggesting that decreased levels of miR-1248 with age may influence the impaired DNA repair capacity that is observed in older individuals [32]. In addition, the mRNAs that were significantly changed in miR-1248 overexpressing cells were imputed into Ingenuity to obtain a miR-1248 putative target list and compared with targets for miR-1248 in TargetScan. There were 18 genes that overlapped between the 2 algorithms and our microarray data (Figure 6E). Two of these potential targets, GAPVD1 and CDK2, we validated were downregulated in miR-1248 overexpressing cells (Figure 6C). Furthermore, the miR-1248 mimic that we have used for overexpression is biotin labeled. Therefore, we performed biotin-labeled precipitations followed by RNA isolation and RT-qPCR to identify mRNAs bound to miR-1248. We found that *CDK2, IL6* and *IL8* mRNAs were enriched in our biotin miR-1248 pulldowns; whereas *GAVD1* mRNA was not (Figure 6F). These data indicate that miR-1248 can bind to *CDK2*, *IL6* and *IL8* mRNAs.

## DISCUSSION

The discovery of miRNAs in circulation has led to important research to determine whether these small noncoding RNAs can be used as diagnostic and prognostic tools for various diseases. Although circulating miRNAs have been examined in diseases such as cancer and cardiovascular disease, the role of miRNAs, in particular circulating miRNAs, in human aging has only begun to be explored. Here, we provide evidence that circulating miRNAs in human serum are differentially expressed with age. Interestingly, we found that several serum miRNAs were significantly decreased with age. Downregulation of miRNAs with human age has been observed by our group and others in human PBMCs and brain as well as in tissues from other species including mice and *C. elegans* [2, 13-15, 17, 33]. Consistent with this idea, we also found that several serum miRNAs significantly decrease in abundance with age in rhesus monkeys. Several studies have also shown that the majority of miRNAs decrease in expression during cellular senescence [6], which models certain aspects of cellular aging. These patterns in expression suggest that levels of miRNAs may decrease with age across species. However, it has also been observed in the mouse brain and liver and during human brain development that specific upregulation of individual miRNAs occurs, which may be important for tissue specific cellular aging or may represent cellular complexi-ty [7, 33]. Nevertheless, it is interesting to note similarities between expression patterns in the different aging models, including our findings here that certain serum miRNAs decrease with human and rhesus monkey age.

The identification of miRNAs in serum is still an emerging field. In order to initially screen miRNAs present in serum *en masse*, we used NGS as this technique provided a genome-wide assessment of expression in young and old individuals and also enabled us to have larger sample groups (11 individuals for each group) that could be assessed at the same time and the ability to assay RNA from serum, which has low concentrations of miRNAs. We validated our initial sequencing results with the more sensitive real-time RT-PCR technique in a larger cohort (n=20 for each group). Based on our sequencing data, we found differential expression of three miRNAs: miR-181a-1, miR-1248 and miR-3607 with human age. In our validation experiments in the larger cohort, we found a significant decrease in expression of miR-151a-3p, miR-181a-5p, and miR-1248. Consistent with our sequencing results, miR-3607-3p and miR-3607-5p were also decreased in our validation cohort, however, there was variability in expression among individuals in this larger cohort and the difference was not significant.

We also examined the expression of miR-21, which has recently been shown to be upregulated in the serum of individuals aged 66-95 [25], however, we did not find any significant differences in either miR-21-3p or miR-21-5p in our young and old cohorts. This may be due to the fact that our young cohort is aged ~30 years and our older cohort is ~64 years, whereas, in the Olivieri study the young group had a broad age range from 20-65 and the older cohort was aged 66-95. In addition, our cohort was made up of individuals of different races and sexes, which could also affect miRNA expression; however, three-way analysis of variance using sex, race and age concluded that race and sex did not significantly correlate with miRNA expression. This suggests that the downregulation of miRNAs that we observed occurs regardless of sex or race.

We used several approaches to gain a better understanding about the putative targets and pathways regulated by the three miRNAs that were significantly decreased with age. First, we analyzed the targets and pathways using both Target Scan and Ingenuity Pathway Analysis. It should be noted that these computational tools have several limitations including that they only predict target sites in the 3'UTR of mRNAs, they often rely on conservation of the miRNA binding site and there is a degree of false positives and negatives associated with prediction analysis of mRNA targets and pathways. Nevertheless, these tools enable the initial assessment of potential targets and pathways, but it is important to further validate experimentally. In agreement with our results showing lower levels with age, all three miRNAs were predicted to be important for organismal survival and development, suggesting that decreased expression may result in the development of age-related phenotypes and age-related diseases. Additionally, all three miRNAs are predicted to be central mediators of inflammatory pathways including NFκB, TNFα and TNF/IL-1/INF-α. Given that the presence of low-grade systemic chronic inflammation is associated with the development and progression of age-related diseases and conditions, it is interesting to speculate that miR-151a-3p, miR-181a-5p and miR-1248 may be important for regulating inflammatory processes and inhibiting inflammation-associated aging in young individuals.

Consistent with this idea, we found that miR-1248 regulates genes involved in various cytokine pathways and specifically modulates the pro-inflammatory cytokines IL-6 and IL-8. In addition, accumulating data indicate that miR-181a-1 targets several inflammatory cytokines including the mRNAs that encode IL-1β, IL-1α, IL-6, IL-8, IL-10, TNFα, TGFβ, FGF2, HMGB1 and LIF [27-29]. Here we found that the levels of pro-inflammatory cytokines IL-6 and TNFα negatively correlated with miR-181a-5p expression and the levels of anti-inflammatory cytokines TGFβ and IL-10 positively correlated with miR-181a-5p. These data suggest that miR-181a-1 expression may be an important age-related factor that regulates inflammatory responses with age. Although in most instances miR-181a-1 attenuates inflammatory responses, in some cases miR-181 family members are upregulated by inflammatory signals and have been reported to be pro-inflammatory, which may explain its reported dual roles as a tumor suppressor-miR or onco-miR in different types of cancers. This may also be explained by the complex role the immune system plays in both inhibiting and promoting cancer initiation, development and progression.

It is interesting to note that in human PBMCs and in the mouse brain miR-181a-1 expression is decreased with age [13, 14, 17, 34] and may also play a role in senescence [35], suggesting that miR-181a-1 may be important for healthy aging. In agreement with this idea, miR-181a has been shown to be upregulated in centenarians [16, 17], who exemplify exceptional longevity. Interestingly, we found that miR-181a-5p expression decreases with age in rhesus monkeys, but increases in extremely old monkeys (n=3, mean age=39.7 years; data not shown). Further suggesting that miR-181a expression may be a potential biomarker of healthy aging in humans and in non-human primates. It should also be noted that miR-181 is an important regulator of hematopoiesis, therefore, decreased miR-181a-1 may impact hematopoietic lineage development during aging [36]. The regulatory role that miR-181a-1 plays in hematopoiesis may also help to explain the high expression we observed in both human and monkey serum.

Our initial screen by NGS indicated that miR-151a/b may be upregulated with age. However, in our more quantitative validation by RT-qPCR in a larger cohort of individuals, we found that miR-151a-3p was significantly downregulated in the serum of old participants. miR-151a-3p has not been well studied, although its expression varies in certain types of cancers and expression has been positively correlated with patient survival [37, 38]. Interestingly, in three different studies of human PBMCs, miR-151a-3p was upregulated in the centenarian populations studied [15, 17] and we found previously that this miRNA was decreased in PBMCs from individuals aged approximately 64 years [14]. Additional work is needed to determine the exact role that miR-151a-3p plays in aging as there may be differences between the old and the oldest old who demonstrate exceptional longevity.

There have been very few published reports on miR-1248. We previously found that miR-1248 expression was decreased in our miRNA screen in PBMCs [14]. In order to better understand the potential role miR-1248 may play in aging, we performed a genome wide microarray and found that miR-1248 regulates the expression of genes that play a role in cytokine and inflammatory pathways, as well as, DNA repair pathways. Although miR-1248 is likely to be involved in various other cellular pathways, these data suggest that decreased serum miR-1248 may potentiate inflammation and DNA damage that occurs with age.

In summary, we have identified miRNAs expressed in the serum of urban, community-dwelling young and old individuals. We found that several miRNAs are decreased with age in both humans and non-human primates, pointing to a potential regulatory role for miRNAs in the aging process. Future work lies ahead in determining the mechanism of this downregulation as well as how to better use miRNAs as diagnostic and therapeutic tools for aging and age-related diseases.

## METHODS

### Study Participants

Fasting blood samples were obtained from participants in the Healthy Aging in Neighborhoods of Diversity across the Life Span (HANDLS) study of the National Institute on Aging Intramural Research Program (NIA IRP), National Institutes of Health (NIH). Samples were collected in vials with no additives, centrifuged and serum was collected, aliquoted and immediately frozen at −80°C until use. HANDLS is a longitudinal, epidemiologic study based in Baltimore, MD that seeks to determine the source of age-associated health disparities by examining the relationship between race, socioeconomic status and health [39]. Participants are Whites and African Americans between the ages of 30-64 at baseline residing in Baltimore, MD. This study has been approved by the Institutional Review Board of the National Institutes of Environmental Health Sciences, NIH and all participants provided written informed consent. The demographics of the cohort used in this study are presented in Table 1. All data presented were collected from participant interviews and self-reported medical histories. For this cohort, we excluded participants with documented Hepatitis B, Hepatitis C or human immunodeficiency virus (HIV) infection. Inflammatory cytokines were measured in serum using SearchLight immunoassays by Aushon Biosystems (Billerica, MA).

### Non-human Primates

Blood samples were collected from anesthetized (Ketamine 7-10 mg/kg, IM or Telazol 3-5 mg/kg, IM) rhesus monkeys (*Macaca mulatta*) after an overnight fast. Serum was aliquoted and immediately frozen at −80°C until use. The monkeys were control animals on NIA IRP studies under protocols approved by the NIA Animal Care and Use Committee. Monkeys were considered healthy at the time of sample collection.

### Next Generation Sequencing

Total RNA was isolated from 400 μL of serum from 11 young (30 yrs) and 11 old (64 yrs) HANDLS participants using Trizol LS (Life Technologies) according to manufacturer's instructions with the addition of a second phenol/chloroform extraction prior to RNA precipitation in order to reduce protein and DNA contamination. Samples were resuspended in 15 μL RNase-free water and frozen at −80°C until further use. In order to identify miRNAs present in serum, we used Illumina's NGS technology. Total RNA samples, as isolated above, were prepared for sequencing using the TruSeq small RNA sample prep kit (Illumina) with multiplexing according to manufacturer's instructions. Briefly, 3' and 5' RNA adapters were ligated to small RNA molecules. RNA was then reverse transcribed and PCR amplified. Prior to amplification, indices to allow for sample multiplexing were incorporated. cDNA was then validated using an Agilent Technologies Bioanalyzer with a high sensitivity DNA chip. Samples in the same multiplex group were pooled and run on a 6% SDS-PAGE gel to purify. After gel purification, small RNA libraries were again validated using an Agilent Technologies Bioanalyzer high sensitivity DNA chip and loaded onto a flow cell for sequencing. Data was analyzed using miRDeep software (v2)[40]. Illumina adapter sequences were clipped and any short sequence tags of length less than 17 bases were removed. miRNA sequences were aligned to human genome (hg19) with no more than 1 base mismatch in the seed region using mirDeep mapper program which uses the popular bowtie algorithm [41]. miRNAs were identified using the MirDeep2 program using human miRBase-19 database [42] of both mature and miRNA precursors. All matches and predicted novel miRNAs were reported and analyzed further by randfold program [43] to identify the folding free energies. Differences in expression of miRNAs identified by sequencing were analyzed in R using Poisson regression.

### Reverse Transcription (RT) followed by real-time, quantitative PCR analysis

Total RNA was isolated from 25 μL of serum from 20 young and 20 old HANDLS participants as well as 10 young and 10 old rhesus monkeys using Trizol LS (Invitrogen) according to the manufacturer's instructions with the following modifications. After precipitation, RNA was re-suspended in 85 μL RNase-free water, 10 μL 10x DNase buffer and 5 μL DNase I (RNase free, Ambion) and incubated at 37 °C for 30 min. 200 μL NT2 buffer [44] and 300 μL Acid:Phenol CHCl_3_ (Ambion) were then added and samples were vortexed for 1 min and centrifuged at room temperature for 5 min. 250 μL of the top layer was extracted and precipitated overnight with ethanol, sodium acetate and GlycoBlue (Ambion) at final concentrations of 70% ethanol, 80 mM sodium acetate and 3×10^−5^ mg/mL GlycoBlue. Samples were then incubated 30 min at 4°C, washed with 75% ethanol, air-dried and resuspended in 15 μL RNase-free water. Samples were frozen at −80°C until further use.

For each participant, 10 μL of RNA was reverse transcribed using the QuantiMir™ cDNA Kit (System Biosciences, Mountain View, CA) according to manufacturer's directions. Real-time RT-PCR reactions were performed using 2x SYBR Green Master Mix (Applied Biosystems) according to the manufacturer's directions on an Applied Biosystems 7500 Real-Time PCR System. In order to amplify cDNA generated from serum samples, we used the Universal Reverse Primer provided by the QuantiMir kit and miRNA specific forward primers designed to match the exact sequences of each miRNA analyzed. We normalized to miR-191 in serum as it was expressed in all participant serum, did not change with age and was the least variable normalization miRNA in all individuals. Data was analyzed using Minitab 16 software.

### Microarray and validation of microarray results

HeLa cells were transfected with 20 nM biotinylated miRIDIAN miR-1248 mimic (Thermo Scientific) or a negative control, biotinylated miRIDIAN *C. elegans* miR-67 (here named Con-miR) using Lipofectamine 2000. Forty eight hours post transfection, total RNA was isolated using the mirVana kit (Invitrogen) according to manufacturer's procedure. Three different experiments were performed and a technical repeat was included for each sample. Samples were analyzed using Illumina's Sentrix Human HT-12 ver4 Expression BeadChips (Illumina, San Diego, CA). Details about the microarray procedure and data analysis are in the Supplemental Methods.

To validate the microarray results, total RNA from the same samples from above (Con-miR and miR-1248 overexpressing) were reverse transcribed using random hexamers and SSII reverse transcriptase (Invitrogen) followed by RT-qPCR using SYBR green PCR master mix (Life Technologies). Sequences used for gene specific primers are in the Supplemental Methods. RT-qPCR was performed on an Applied Biosystems 7500 Real-Time PCR System.

### Immunoprecipitation of mRNAs associated with biotinylated-miR-1248

HeLa cells were transfected with 20 nM biotinylated miR-1248 (miR-1248) or a negative control, biotinylated *C. elegans* miR-67 (Con-miR) using Lipofectamine 2000. Twenty-four hours post transfection, cells were lysed in buffer containing 20 mM Tris-HCl (pH 7.5), 100 mM KCl, 5 mM MnCl_2_, 0.3% NP-40, 50 U RNase Out (Invitrogen) and a protease inhibitor cocktail (Roche Applied Science). Samples were then centrifuged for 10 minutes at 4°C at 10,000 × *g*. After a 2 hour pre-incubation of Streptavidin Dynabeads in lysis buffer containing BSA (1 mg/mL) and yeast tRNA (1 mg/mL), cytoplasmic lystates were applied to beads and incubated at 4°C for 4 hours with rotation. Samples were washed five times with lysis buffer after which, RNA bound to beads was isolated using Trizol LS according to manufacturer's instructions with the following modifications: after RNA precipitation, RNA was DNase treated for 30 min at 37°C and then extracted with Acid:Phenol CHCl_3_ (Ambion) before a second precipitation with 100% Ethanol. RNA pellets were re-suspended in 15 μL water. mRNAs associating with biot-miR-1248 were analyzed using gene specific primers (see Supplemental Methods) followed by RT-qPCR.

## SUPPLEMENTAL METHODS, REFERENCES AND TABLE



## References

[R1] Tacutu R, Craig T, Budovsky A, Wuttke D, Lehmann G, Taranukha D, Costa J, Fraifeld VE, de Magalhaes JP (2013). Human Ageing Genomic Resources: integrated databases and tools for the biology and genetics of ageing. Nucleic Acids Res.

[R2] Smith-Vikos T, Slack FJ (2012). MicroRNAs and their roles in aging. Journal of cell science.

[R3] Jung HJ, Suh Y (2012). MicroRNA in Aging: From Discovery to Biology. Current genomics.

[R4] Leung AK, Sharp PA (2010). MicroRNA functions in stress responses. Molecular cell.

[R5] Garzon R, Calin GA, Croce CM (2009). MicroRNAs in Cancer. Annual review of medicine.

[R6] Gorospe M, Abdelmohsen K (2011). MicroRegulators come of age in senescence. Trends in Genetics.

[R7] Bates DJ, Liang R, Li N, Wang E (2009). The impact of noncoding RNA on the biochemical and molecular mechanisms of aging. Biochimica et biophysica acta.

[R8] Bartel DP, Chen CZ (2004). Micromanagers of gene expression: the potentially widespread influence of metazoan microRNAs. Nat Rev Genet.

[R9] Boehm M, Slack F (2005). A developmental timing microRNA and its target regulate life span in C. elegans. Science.

[R10] de Lencastre A, Pincus Z, Zhou K, Kato M, Lee SS, Slack FJ (2010). MicroRNAs both promote and antagonize longevity in C. elegans. Curr Biol.

[R11] Ibanez-Ventoso C, Yang M, Guo S, Robins H, Padgett RW, Driscoll M (2006). Modulated microRNA expression during adult lifespan in Caenorhabditis elegans. Aging cell.

[R12] Pincus Z, Smith-Vikos T, Slack FJ (2011). MicroRNA predictors of longevity in Caenorhabditis elegans. PLoS genetics.

[R13] Inukai S, de Lencastre A, Turner M, Slack F (2012). Novel microRNAs differentially expressed during aging in the mouse brain. PloS one.

[R14] Noren Hooten N, Abdelmohsen K, Gorospe M, Ejiogu N, Zonderman AB, Evans MK (2010). microRNA expression patterns reveal differential expression of target genes with age. PloS one.

[R15] ElSharawy A, Keller A, Flachsbart F, Wendschlag A, Jacobs G, Kefer N, Brefort T, Leidinger P, Backes C, Meese E, Schreiber S, Rosenstiel P, Franke A (2012). Genome-wide miRNA signatures of human longevity. Aging cell.

[R16] Gombar S, Jung HJ, Dong F, Calder B, Atzmon G, Barzilai N, Tian XL, Pothof J, Hoeijmakers JH, Campisi J, Vijg J, Suh Y (2012). Comprehensive microRNA profiling in B-cells of human centenarians by massively parallel sequencing. BMC genomics.

[R17] Serna E, Gambini J, Borras C, Abdelaziz KM, Belenguer A, Sanchis P, Avellana JA, Rodriguez-Manas L, Vina J (2012). Centenarians, but not octogenarians, up-regulate the expression of microRNAs. Scientific reports.

[R18] Cortez MA, Bueso-Ramos C, Ferdin J, Lopez-Berestein G, Sood AK, Calin GA (2012). MicroRNAs in body fluids--the mix of hormones and biomarkers. Nat Rev Clin Oncol.

[R19] Brase JC, Wuttig D, Kuner R, Sultmann H (2010). Serum microRNAs as non-invasive biomarkers for cancer. Mol Cancer.

[R20] Reid G, Kirschner MB, van Zandwijk N (2011). Circulating microRNAs: Association with disease and potential use as biomarkers. Critical reviews in oncology/hematology.

[R21] Dorn GW (2010). MicroRNAs in cardiac disease. Transl Res.

[R22] Li C, Fang Z, Jiang T, Zhang Q, Liu C, Zhang C, Xiang Y (2013). Serum microRNAs profile from genome-wide serves as a fingerprint for diagnosis of acute myocardial infarction and angina pectoris. BMC Med Genomics.

[R23] Liu Z, Zhou C, Liu Y, Wang S, Ye P, Miao X, Xia J (2012). The expression levels of plasma micoRNAs in atrial fibrillation patients. PloS one.

[R24] Ji X, Takahashi R, Hiura Y, Hirokawa G, Fukushima Y, Iwai N (2009). Plasma miR-208 as a biomarker of myocardial injury. Clinical chemistry.

[R25] Olivieri F, Spazzafumo L, Santini G, Lazzarini R, Albertini MC, Rippo MR, Galeazzi R, Abbatecola AM, Marcheselli F, Monti D, Ostan R, Cevenini E, Antonicelli R (2012). Age-related differences in the expression of circulating microRNAs: miR-21 as a new circulating marker of inflammaging. Mechanisms of ageing and development.

[R26] Roth GS, Mattison JA, Ottinger MA, Chachich ME, Lane MA, Ingram DK (2004). Aging in rhesus monkeys: relevance to human health interventions. Science.

[R27] Hutchison ER, Kawamoto EM, Taub DD, Lal A, Abdelmohsen K, Zhang Y, Wood WH, Lehrmann E, Camandola S, Becker KG, Gorospe M, Mattson MP (2013). Evidence for miR-181 involvement in neuroinflammatory responses of astrocytes. Glia.

[R28] Wu C, Gong Y, Yuan J, Zhang W, Zhao G, Li H, Sun A, Zou Y, Ge J (2012). microRNA-181a represses ox-LDL-stimulated inflammatory response in dendritic cell by targeting c-Fos. Journal of lipid research.

[R29] Xie W, Li M, Xu N, Lv Q, Huang N, He J, Zhang Y (2013). miR-181a Regulates Inflammation Responses in Monocytes and Macrophages. PloS one.

[R30] Li X, Mai J, Virtue A, Yin Y, Gong R, Sha X, Gutchigian S, Frisch A, Hodge I, Jiang X, Wang H, Yang X-F (2012). IL-35 Is a Novel Responsive Anti-inflammatory Cytokine A New System of Categorizing Anti-inflammatory Cytokines. PloS one.

[R31] Saraiva M, O'Garra A (2010). The regulation of IL-10 production by immune cells. Nat Rev Immunol.

[R32] Jacob KD, Noren Hooten N, Trzeciak AR, Evans MK (2013). Markers of oxidant stress that are clinically relevant in aging and age-related disease. Mechanisms of ageing and development.

[R33] Persengiev S, Kondova I, Otting N, Koeppen AH, Bontrop RE (2011). Genome-wide analysis of miRNA expression reveals a potential role for miR-144 in brain aging and spinocerebellar ataxia pathogenesis. Neurobiology of aging.

[R34] Li G, Yu M, Lee WW, Tsang M, Krishnan E, Weyand CM, Goronzy JJ (2012). Decline in miR-181a expression with age impairs T cell receptor sensitivity by increasing DUSP6 activity. Nature medicine.

[R35] Mancini M, Saintigny G, Mahe C, Annicchiarico-Petruzzelli M, Melino G, Candi E (2012). MicroRNA-152 and −181a participate in human dermal fibroblasts senescence acting on cell adhesion and remodeling of the extra-cellular matrix. Aging.

[R36] O'Connell RM, Baltimore D, Eran H (2012). Chapter six – MicroRNAs and Hematopoietic Cell Development. Current Topics in Developmental Biology.

[R37] Hui AB, Lin A, Xu W, Waldron L, Perez-Ordonez B, Weinreb I, Shi W, Bruce J, Huang SH, O'Sullivan B, Waldron J, Gullane P, Irish JC (2013). Potentially prognostic miRNAs in HPV-associated oropharyngeal carcinoma. Clin Cancer Res.

[R38] McNally ME, Collins A, Wojcik SE, Liu J, Henry JC, Jiang J, Schmittgen T, Bloomston M (2013). Concomitant dysregulation of microRNAs miR-151-3p and miR-126 correlates with improved survival in resected cholangiocarcinoma. HPB.

[R39] Evans MK, Lepkowski JM, Powe NR, LaVeist T, Kuczmarski MF, Zonderman AB (2010). Healthy aging in neighborhoods of diversity across the life span (HANDLS): overcoming barriers to implementing a longitudinal, epidemiologic, urban study of health, race, and socioeconomic status. Ethnicity & disease.

[R40] Friedlander MR, Chen W, Adamidi C, Maaskola J, Einspanier R, Knespel S, Rajewsky N (2008). Discovering microRNAs from deep sequencing data using miRDeep. Nature biotechnology.

[R41] Langmead B, Trapnell C, Pop M, Salzberg SL (2009). Ultrafast and memory-efficient alignment of short DNA sequences to the human genome. Genome biology.

[R42] Kozomara A, Griffiths-Jones S (2011). miRBase: integrating microRNA annotation and deep-sequencing data. Nucleic Acids Res.

[R43] Bonnet E, Wuyts J, Rouze P, Van de Peer Y (2004). Evidence that microRNA precursors, unlike other non-coding RNAs, have lower folding free energies than random sequences. Bioinformatics.

[R44] Noren Hooten N, Kompaniez K, Barnes J, Lohani A, Evans MK (2011). Poly(ADP-ribose) polymerase 1 (PARP-1) binds to 8-oxoguanine-DNA glycosylase (OGG1). J Biol Chem.

