# Supplementary material for: Age-related changes in microRNA levels in serum
Source: Aging (Albany NY). 2013 Sep 27;5(10):725–40. doi: 10.18632/aging.100603 (PMC3838776; doi:10.18632/aging.100603)
Supplement: Supplementary file 1 [file aging-05-725-s001.pdf]

## SUPPLEMENTAL METHODS

<u>Illumina oligonucleotide microarray.</u> Transcrip-tional profiling of Con-miR and miR-1248 overexpressing cells was performed using Illumina Sentrix BeadChips. Total RNA (isolated as in the Methods) was used to generate biotin-labeled cRNA using the Illumina TotalPrep RNA Amplification Kit. In brief, 0.5□g of total RNA was first converted into single-stranded cDNA with reverse transcriptase using an oligo-dT primer containing the T7 RNA polymerase promoter site and then copied to produce double-stranded cDNA molecules. The double stranded cDNA was cleaned and concentrated with the supplied columns and used in an overnight *in vitro* transcription reaction in order to generate single-stranded RNA (cRNA) incorporating biotin-16-UTP. A total of 0.75□g of biotin-labeled cRNA was hybridized at 58$^{o}$C for 16 hours to Illumina's Sentrix Human HT-12 ver4 Expression BeadChips (Illumina, San Diego, CA). Each BeadChip has approximately 48,000 transcripts with about 15-fold redundancy. Next, the arrays were washed, blocked and the labeled cRNA was detected by staining with streptavidin-Cy3. Hybridized arrays were scanned using an Illumina BeadStation 500X Genetic Analysis Systems scanner and the image data extracted using the Illumina GenomeStudio software, version 1.9.0. For statistical analysis, the expression data were filtered to include only probes with a consistent signal on each chip; the probe original signal filter value was established at detection *p* value < 0.02. The complete normalized and raw dataset have been submitted to GEO (Accession Number; GSE51309). The resulting dataset was analyzed as described below.

<u>Microarray data analysis.</u> Data from microarray was analyzed using DIANE 6.0,a spreadsheet-based microarray analysis program based on SAS JMP7.0 system. First, raw microarray data were subjected to filtering by the detection p-value and Z normalization and the data are further tested for significant changes as previously described (Cheadle *et al.* 2003). The sample quality was initially analyzed using scatter plots, principal component analysis, and gene sample Zscores based on hierarchy clustering to exclude possible outliners. ANOVA tests were used to eliminate the genes with larger variances within each comparing group. Genes were determined to be differentially expressed after calculating the Z ratio, which indicates the fold-difference between experimental groups, and false discovery rate (fdr), which controls for the expected proportion of false rejected hypotheses. Significant changes in individual genes were considered if the p value $\leq$0.05, absolute value of Z ratio $\geq$ 1.5 and fdr $\leq$ 0.3. A total of 3510 differentially expressed genes were identified as significantly changed in miR-1248 overexpressing cells compared to Con-miR. Hierarchy clustering/K-means clustering and Principal Components Analysis (PCA) were performed to identify clustering within the two groups. Array data for each experimental sample was also originally hierarchically clustered in Ilumina Bead Studio version 2.0.

We employed the Parametric Analysis of Gene-set Enrichment (PAGE) algorithm for gene set enrichment analysis by using all of the genes in each sample as input against and the data set supplied by Gene Ontology Institute and MIT Broad Institute (De *et al.* 2010). For comparisons between Con miRNA and miR-1248 samples, the lists of differentially expressed genes and Z ratios were entered into the PAGE Pathway Analysis software to organize them according to known biological pathways. The Enrichment Zscores for each functional grouping were calculated based on the chance of mRNA abundance changes predicting these interactions and networks by z-test. The P-value was calculated by comparing the number of user-specified genes of interest participating in a given function or pathway relative to the total number of occurrences of these genes in all functional/pathway annotations stored in the knowledge base. All of the Pathways must at least have three genes found in the microarray gene set. The p value $\leq$0.05 and fdr $\leq$0.3 are the cutoff criteria for the significant pathway selection. The canonical pathway results, sorted by Z ratio, are represented by a heat map in Fig. 6D. The top 25 downregulated pathways are shown in Fig. 6D and the entire heat map of all significant canonical pathways is shown in Supporting Fig. 1.

<u>Gene specific primers.</u> The oligomer pairs (forward and reverse) indicated below were used for real time RT-PCR for each gene:
GGCCACAGCTGCCTCTTC and CCAGCAGATTCCATACCAATGA for ACTC1
GGCTAAGAGGAGCTGATTCGTTATC and AGAGATTGGGTTACAGGGACGTAT for C1orf116
CTCCCCTGGATGAAGATGGA and GCTGCCTTGGCCGAAAT for CDK2
GCTCCTCCTGTTCGACAGTCA and ACCTTCCCCATGGTGTCTGA for GAPDH
GGCTGCACCTCATTCATCATC and TCATCGCTATCTTTGCGTTCTTC for GAVD1
CCGGGAACGAAAGAGAAGCT and GCGCTTGTGGAGAAGGAGTT for IL-6
CTTTCCACCCCAAATTTATCAAAG and CAGACAGAGCTCTCTTCCATCAGA for IL-8
CGCCAGCGATCATGTCTACA and CTCCATCCCGAGTGCAGAAT for LYPD3

GAACTGCTGGAAGGAGACTGGAT and TTCCGGTTGAAGATTTGACAA for TMX1 CGTGAAGGAGTACGTGAATGCT and GGCGAATGAGTCCTCAATGC for ZNF185.

## REFERENCES


Cheadle C, Vawter MP, Freed WJ , Becker KG. Analysis of microarray data using Z score transformation. The Journal of molecular diagnostics : JMD. 2003; 5:73-81.

De S, Zhang Y, Garner JR, Wang SA , Becker KG. Disease and phenotype gene set analysis of disease-based gene expression in mouse and human. Physiological genomics. 2010; 42A:162-167.


## SUPPLEMENTARY TABLE

Please browse full text version of this manuscript to see the the Supplemental **Table S1.** Differentially expressed genes that are significantly changed by miR-1248 overexpression.

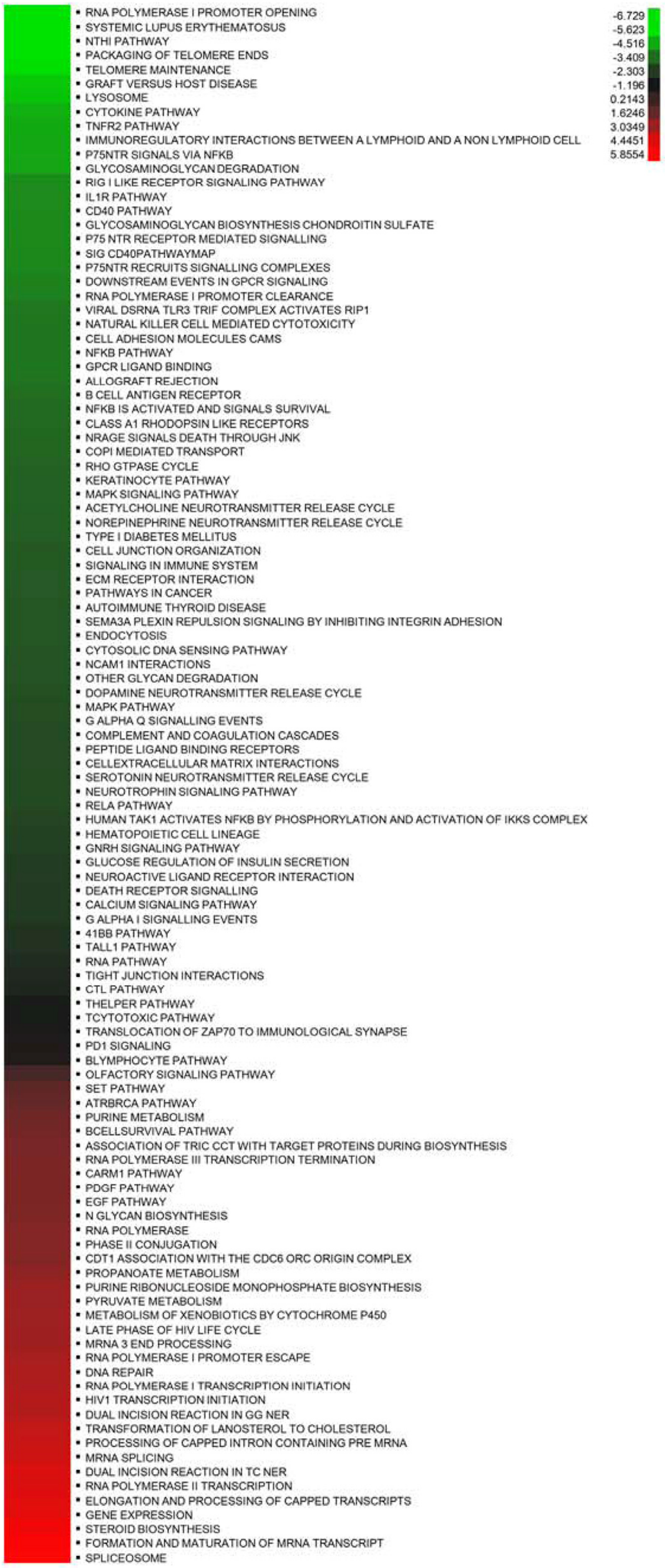


**Figure S1. Significant canonical pathways regulated by miR-1248 expression.** miR-1248 was overexpressed in HeLa cells as described in Methods. Total RNA from Con-miR and miR-1248 expressing cells were analyzed using microarray as in Methods and Supplemental Methods. Analysis was performed as in Supplemental Methods and the significant canonical pathways regulated by miR-1248 are shown by heat map. Pathways were sorted by Z ratio.
